# *Burkholderia semiarida* as Cause of Recurrent Pulmonary Infection in Immunocompetent Patient, China

**DOI:** 10.3201/eid3006.231676

**Published:** 2024-06

**Authors:** Dai Kuang, Feng Liu, Shen Tian, Wei Liu, Anyang Li, Yujing Zhou, Huaping Huang, Qianfeng Xia

**Affiliations:** NHC Key Laboratory of Tropical Disease Control, School of Tropical Medicine, Hainan Medical University, Haikou, China (D. Kuang, S. Tian, W. Liu, A. Li, Y. Zhou, Q. Xia);; The First Affiliated Hospital of Hainan Medical University, Haikou (F. Liu, H. Huang)

**Keywords:** pneumonia, bacteria, antimicrobial resistance, *Burkholderia*, *Burkholderia semiarida*, recurrent infection, immunocompetent patient, China

## Abstract

*Burkholderia semiarida* was previously identified solely as a plant pathogen within the *Burkholderia cepacia* complex. We present a case in China involving recurrent pneumonia attributed to *B*. *semiarida* infection. Of note, the infection manifested in an immunocompetent patient with no associated primary diseases and endured for >3 years.

*Burkholderia* *semiarida* has only been reported as a plant pathogen causing onion sour skin (*1*). The *Burkholderia* genus encompasses >120 bacterial species that are typically reported to inhabit soil and water environments (*2*). The *Burkholderia* species that are most frequently reported to cause infection in humans are *B*. *cenocepacia*, *B*. *mallei*, and *B*. *pseudomallei*. However, severe infections have been also caused by other species (*3*,*4*). We report a rare case of *B*. *semiarida* human infection: recurrent pneumonia in an immunocompetent patient. Ethics approval for this study, including the waiver of informed consent of the clinical strains and samples, was approved by the Ethics Committee of the First Affiliated Hospital of Hainan Medical University under approval no. 2023-KYL-219.

## The Study

A 56-year-old woman was admitted to The First Affiliated Hospital of Hainan Medical University (Haikou, China) on February 23, 2022. She reported a 3-year history of recurrent cough, copious sputum production, and chest pain, which had worsened over the previous 5 days. 

On August 18, 2020, the woman had been admitted to Hainan Traditional Chinese Medicine Hospital for a cough without obvious cause that was accompanied by white sticky sputum and right-sided chest pain that was aggravated by coughing. She did not have fever or hemoptysis. A computed tomography (CT) scan of the chest showed infiltrative lesions in the right lung (Figure, panel A). Initial laboratory workup revealed leukocyte count of 11.1 × 10^9^/L with 8.11 × 10^9^/L neutrophils. Symptoms resolved substantially after 10 days of treatment with ceftriaxone/tazobactam and levofloxacin.

**Figure:**
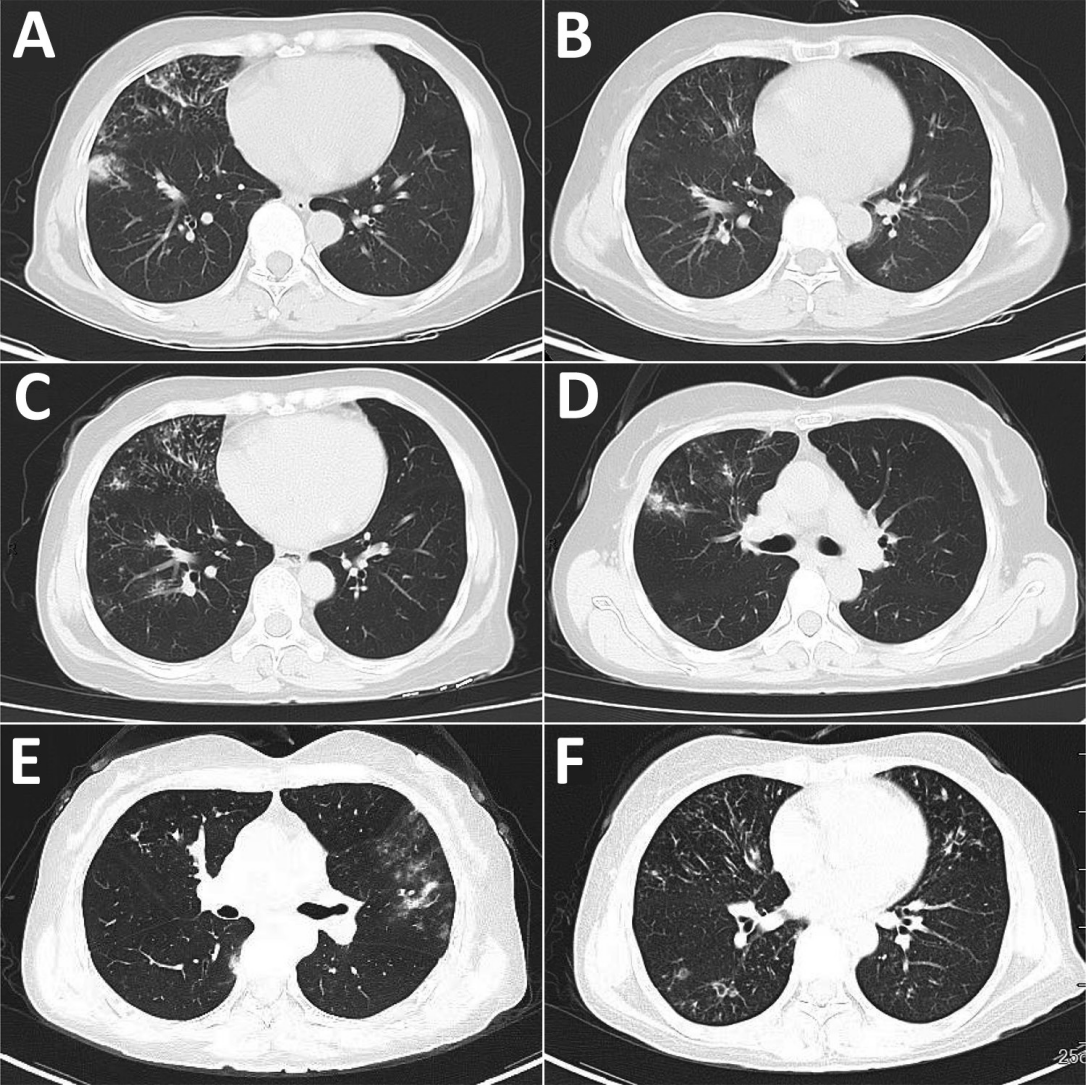
Computed tomography scans of the chest at different stages of disease in immunocompetent patient with recurrent pulmonary infection caused by *Burkholderia semiarida*, China. A) Infiltrative lesions in the right lung in August 2020; B) multiple infiltrative lesions in both lungs substantially resorbed in June 2021; C) multiple infiltrative lesions of both lungs, predominantly in the right lung, in February 2022; D) bronchiectasis with scattered multiple lesions in both lungs and increased exudative lesions in the right upper lung in May 2022; E) lesions in the right lung more resorbed than before and multiple emerging lesions in the left lung in August 2022; F) lesions in the left lung more absorbed than before, with multiple emerging foci in the right lung in March 2023.

The patient was readmitted to the hospital on November 3, 2020, with a recurrence of the symptoms previously described. Chest CT scans showed infiltrative lesions in both lungs and new lesions in the left upper and lower lobes. Results of routine blood tests were within normal limits, albeit with a slightly elevated hypersensitive C-reactive protein (6.64 mg/L) and erythrocyte sedimentation rate (29 mm/h). Metagenomic next-generation sequencing (mNGS) analysis of bronchoalveolar lavage fluid (BALF) suggested that the infection could have resulted from *B*. *cepacia*. The patient received a 10-day intravenous meropenem treatment, followed by 8 weeks of trimethoprim/sulfamethoxazole. A subsequent chest CT showed mild bronchiectasis in the right middle lobe and lower lingula segment of the left upper lobe, in addition to multiple lesions. *B. cenocepacia* infection was suspected, according to mNGS retesting on January 27, 2021. Thus, the ongoing trimethoprim/sulfamethoxazole treatment was extended to 24 weeks. CT chest scans on June 7, 2021, showed multiple lesions in both lungs that were substantially resorbed, whereas new lesions emerged in right middle lobes (Figure, panel B). Treatment was then changed to minocycline for another 4 weeks. The patient returned to the hospital on July 20, 2021, because of recurrent right-sided chest pain. Chest CT showed more serious lesions in both lungs, and BALF culture suggested *B. cenocepacia* infection. The isolates were sensitive to ceftazidime, meropenem, trimethoprim/sulfamethoxazole, minocycline, and levofloxacin. Treatment was then adjusted to intravenous meropenem and 20 weeks of amoxicillin/clavulanate potassium.

On February 20, 2022, the previous symptoms recurred, accompanied by hemoptysis. Chest CT scan revealed multiple infiltrative lesions, predominantly in the right lung (Figure, panel C). The patient’s personal and family medical history were unremarkable. Physical examination showed normal temperature, coarse breath sounds, moist rales, and no enlargement of superficial lymph nodes, lower extremity swelling, or heart and abdominal abnormalities. Results of tests of complete blood count, C-reactive protein, procalcitonin, and liver and kidney function were within reference ranges. Tests for tumor markers and extractable nuclear antigen were negative. Peripheral blood T lymphocyte subsets, natural killer cells, B lymphocytes, serum immunoglobulins, and complement levels were all within reference ranges. Gram staining demonstrated the agents as gram-negative rods or cocci. Tests for mycobacteria and fungi were negative.

After a week of intravenous imipenem/cilastatin treatment, the patient was discharged with improved symptoms. Outpatient therapy included oral nemonoxacin and amoxicillin/clavulanate, along with acetylcysteine and subcutaneous thymopentin. A follow-up chest CT in May 2022 revealed mild bronchiectasis and multiple lesions in both lungs, including increased exudative lesions in the right upper lung (Figure, panel D). Subsequently, the therapy was adjusted to moxifloxacin and doxycycline. Chest CT scan in August indicated better absorption of right lung lesions but new infections in the left lung (Figure, panel E). The therapy was then adjusted to oral ciprofloxacin with doxycycline. The mNGS conducted in October indicated the possibility of *B. anthina* infection. Chest CT scan in March 2023 showed improved left lung lesions but new infection foci in the right lung (Figure, panel F). At the time of this article, the patient was still receiving conservative outpatient treatment. 

BALF samples were sent to Hainan Medical University for bacterial isolation and characterization. The suspected bacteria showed typical morphologic characteristics of *B. cepacia* complex species (Appendix Figure 1). Susceptibility tests showed sensitivity to ceftazidime, meropenem, and trimethoprim/sulfamethoxazole, intermediate resistance to minocycline, and resistance to levofloxacin. Whole-genome sequencing indicated 3 circular chromosomes and 1 plasmid with guanine-cytosine content of 66.89% (National Center for Biotechnology Information Bioproject accession no. PRJNA1028481). The values of digital DNA-DNA hybridization (*5*) and average nucleotide identity (*6*) were used to compare the representative genomes of *Burkholderia* genus in the National Center for Biotechnology Information Reference Sequence database. Pairwise comparison showed that digital DNA-DNA hybridization and average nucleotide identity values between our strain with representative genome of *B. semiarida* CCRMBC171 (accession no. GCF_029268915.1) were 88.8% and 98.6%, which are above the threshold of 70% and 95%–96% used for bacterial species delineation (*6*,*7*) (Appendix Figure 2). Thus, although misidentification can occur using mNGS to distinguish *B. semiarida* from other *Burkholderia* species, we can conclude that the recurrent pulmonary infection was caused by *B.* *semiarida*.

## Conclusions

We present a rare case of pulmonary infection caused by *B. semiarida* in a 56-year-old immunocompetent woman. The early clinical manifestations were cough, sputum production, and chest pain. Hemoptysis and moist crackles in the lungs emerged late in the course of disease. The inflammatory markers and immune indicators suggest the patient had normal immune function. Chest CT scans indicated inflammation alternated in both lungs for >3 years. Mild local bronchiectasis occurred repeatedly at multiple sites on both sides. Persistent infections usually result in high levels of illness and death (*8*). Thus, a long period of follow-up after hospitalization should be used to assess the outcome of therapy in infections caused by *B. semiarida*.

The choice of antimicrobial therapy is usually made on the basis of in vitro susceptibility. In our case, the infection failed to respond to prolonged antibiotic therapy using common medications despite apparent drug susceptibility. Of note, the strain exhibited a decreasing susceptibility to minocycline and levofloxacin as the disease endured and progressed. This observation suggests that the frequency of antibiotic use might drive rapid evolutionary adaptation of resistance as previously described (*9*,*10*). This case report highlights that patients who have persistent respiratory symptoms who do not respond to initial treatment might be candidates for closer assessment for *Burkholderia* infection.

In summary, we present the clinical features of a patient with *B. semiarida* infection. Key aspects of this case were the occurrence of infection in an immunocompetent patient with no related primary diseases, that inflammation alternated in both lungs and persisted for >3 years, and that the infection did not appear to respond to in vitro active antimicrobial medications. Our case report alerts *B. semiarida* as a stubborn pathogen in persistent pulmonary infection and highlights the importance of extended follow-up care to assess the outcome of antimicrobial therapy in *Burkholderia* infections.

Appendix Additional information about *Burkholderia semiarida* as cause of recurrent pulmonary infection in immunocompetent patient, China
