# Supplementary material for: Burkholderia semiarida as Cause of Recurrent Pulmonary Infection in Immunocompetent Patient, China
Source: Emerg Infect Dis. 2024 Jun;30(6):1249–52. doi: 10.3201/eid3006.231676 (PMC11138970; doi:10.3201/eid3006.231676)
Supplement: Appendix — Additional information about Burkholderia semiarida as cause of recurrent pulmonary infection in immunocompetent patient, China [file 23-1676-Techapp-s1.pdf]

Article DOI: https://doi.org/10.3201/eid3006.231676

*EID cannot ensure accessibility for Supplemental Materials supplied by authors. Readers who have difficulty accessing supplementary content should contact the authors for assistance.*

# *Burkholderia semiarida* as Cause of Recurrent Pulmonary Infection in Immunocompetent Patient, China

**Appendix.**

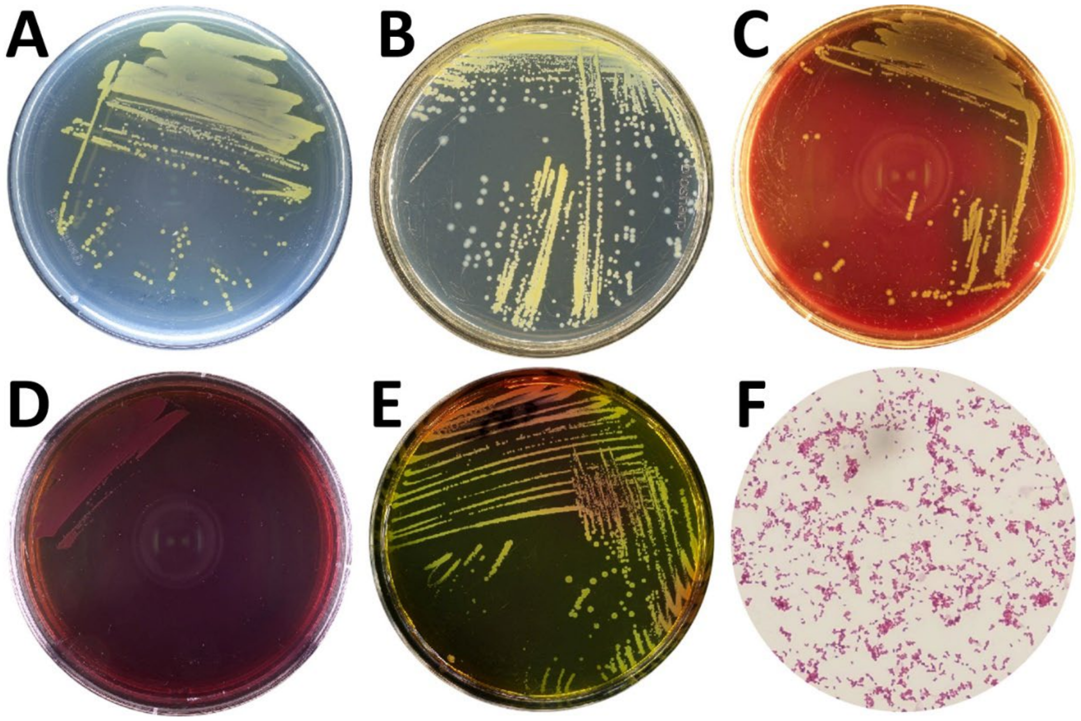


**Appendix Figure 1.** Colonial morphology of *Burkholderia semiarida* HNBS001 shown in LB agar (A), TSA agar (B), blood agar (C), MacConkey agar (D) and *Burkholderia cepacia* selective agar (E); Gram stain of *Burkholderia semiarida* HNBS001 (F). Bacteria growing on LB agar and blood agar media showed colonies with yellow, rounded shapes, smooth edges, and convex elevation, whereas

on MacConkey agar were pink, small, smooth, with sheen and convex and on *Burkholderia cepacia* selective agar were yellowish-greenish-brown or surrounded by white colonies tinged with pink. The bacterial cells were gram-negative bacilli shapes with pink cell color based on the results of the gram stain.

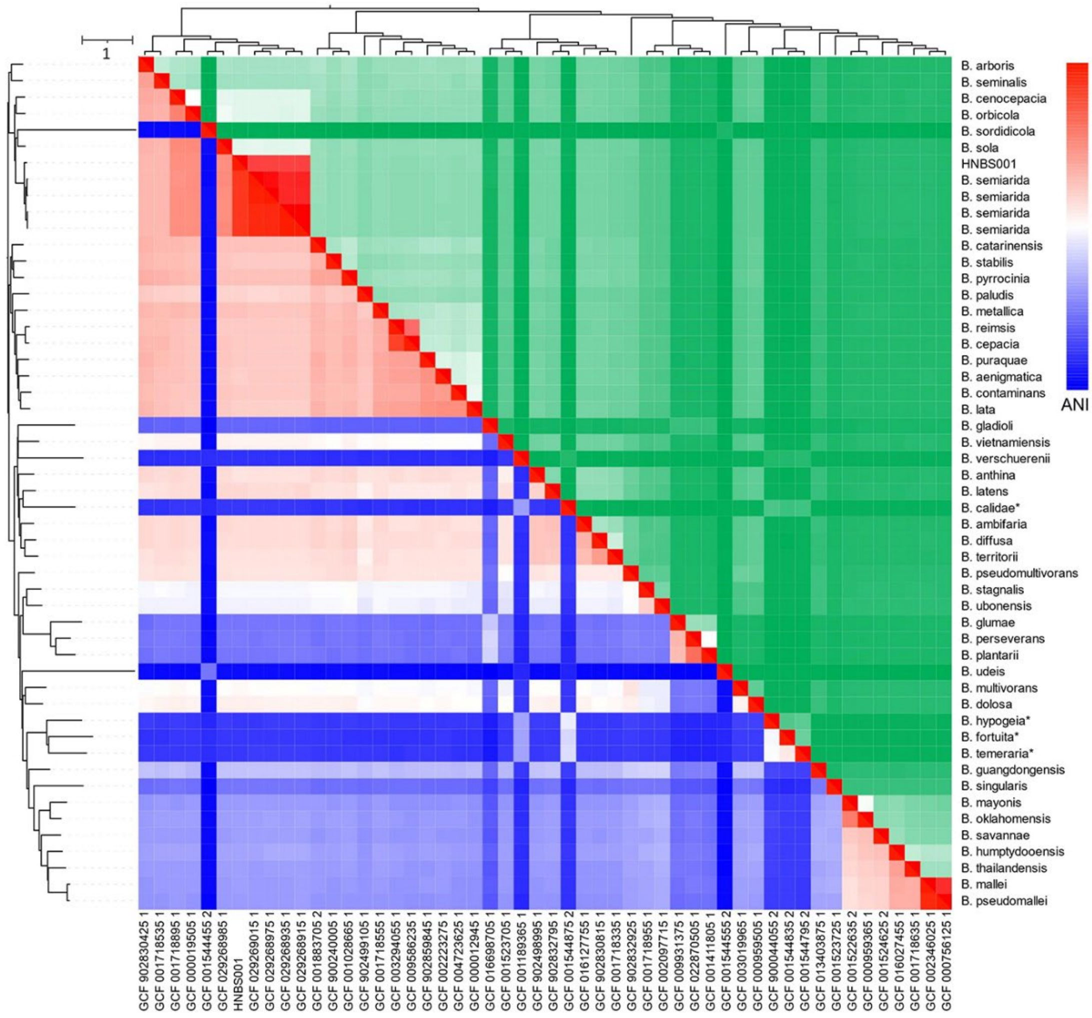


**Appendix Figure 2.** Phylogenetic tree and pairwise comparisons based on both dDDH (d4) and ANI values of HNBS001 and representative genomes of *Burkholderia*. The tree was inferred with fasttree (*1*) from SNPs matrix calculated from genome sequences by kSNP (*2*). The tree was rooted at the midpoint. The same tree clustering was used for both rows (labeled with *Burkholderia* species name) and columns (ignored branch lengths, labeled with accession number) in the heatmap. The strain we

isolated in this study was named as HNBS001. The genome of 48 representative *Burkholderia* species and three other *B. semiarida* were downloaded from the Refseq database accessed on September 26, 2023. Cutoff values for species clustering was 70% for dDDH (d4) and 96% for ANI. The asterisk represents the species were suggested transfer to the genus *Caballeronia* (*3*).

**References**


1. Price MN, Dehal PS, Arkin AP. FastTree 2—approximately maximum-likelihood trees for large alignments. PLoS One. 2010;5:e9490. PubMed https://doi.org/10.1371/journal.pone.0009490

2. Gardner SN, Slezak T, Hall BG. kSNP3.0: SNP detection and phylogenetic analysis of genomes without genome alignment or reference genome. Bioinformatics. 2015;31:2877–8. PubMed https://doi.org/10.1093/bioinformatics/btv271

3. Dobritsa AP, Linardopoulou EV, Samadpour M. Transfer of 13 species of the genus Burkholderia to the genus Caballeronia and reclassification of Burkholderia jirisanensis as Paraburkholderia jirisanensis comb. nov. Int J Syst Evol Microbiol. 2017;67:3846–53. PubMed https://doi.org/10.1099/ijsem.0.002202
